# Comparison of taste components in stewed beef broth under different conditions by means of chemical analyzed

**DOI:** 10.1002/fsn3.1376

**Published:** 2020-01-09

**Authors:** Linhan Wang, Kaina Qiao, Wen Duan, Yuyu Zhang, Junfei Xiao, Yan Huang

**Affiliations:** ^1^ Beijing Advanced Innovation Center for Food Nutrition and Human Health Beijing Laboratory for Food Quality and Safety, Beijing Technology & Business University (BTBU) Beijing China

**Keywords:** 5′‐nucleotide, amino acid, organic acid, stewed beef broth, taste compound

## Abstract

The aims of this study were to investigate the effect of stewing process on the content of taste compounds in stewing beef broth. The amino acids, 5′‐nucleotides, and organic acids in stewing beef broth were determined by HPLC. The results showed that the contents of four 5′‐nucleotides in raw beef were significantly lower than that in stewed beef broth. The addition of spices, salt, and sucrose was beneficial to promote the release of amino acid in beef broth. The highest contents of umami, sweet amino acid, and total amino acid were 907.67, 2930.11, and 5088.76 μg/g in stewed beef broth with salt addition, and 1085.10, 3367.48, and 5595.20 μg/g with sucrose addition. The contents of those in the stewed beef optimal group (s‐b‐o) were 7008.53, 34007.67, and 49282.82 μg/g, respectively, which was far higher than that with salt addition and sucrose addition. The content of total amino acid and total organic acid was significantly higher in s‐b‐o‐o than in s‐b‐o. The proper amount of blend oil was beneficial to the release of flavor substances in stewed beef broth. The EUC value of the stewed beef blank group (s‐b‐b) was 3.50 g MSG/100 g. The addition of spices could significantly increase the EUC of stewed beef broth. The TAVs of 8 compounds were more than 1 in the sample of s‐b‐o‐o, including Asp, Glu, Pro, Ala, Val, Met, Arg, and tartaric acid. These 8 compounds contribute more to the taste of stewed beef broth.

## Graphical abstract

To investigate the stewing process effect on taste content in stewing beef broth, the amino acids, 5′‐nucleotides, and organic acids were determined by HPLC. The addition of spices, salt, oil and sucrose promoted the release of amino acid. The TAVs of 8 compounds were more than 1 in the sample of s‐b‐o‐o.

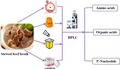

## 1 INTRODUCTION

Beef is one of the most commonly consumed meats worldwide, and its consumption continues to increase. Beef is rich in high‐quality protein, minerals, and other nutrients. In China, beef cuts are very popular and widely consumed. It is commonly used for preparing traditional dishes such as Minced Beef Thick Soup, Stewed Beef Brisket with Tomato, Stewed Beef with Carrots, Stewed Beef in Casserole, Stewed Beef Brisket with Radish in Casserole, Boiled Beef Slices, and beef broth combined with various food ingredients (Hong, Kim, Lee, & Heo, 2013; Kranz et al., 2018). Beef broth is particularly popular for its palatable taste.

Flavor represents one of the most important quality attributes contributing to the widespread consumption of the broth (Jayasena & others, 2015). Liebig first isolated and identified the precursor compound of umami as inosinic acid from beef soup in 1847 (Brosnan & Brosnan, 2009). Kranz et al., (2018) quantitatively analyzed the basic taste compounds in a traditional vegetable beef soup by HPLC. The detected 5′‐nucleotides, amino acids, organic acids, and inorganic salts in beef soup were as follows: hypoxanthine, xanthine, creatine, creatinine, taurine, inosine, bitter and sweet amino acids, glutamine, glutamate, asparagine, aspartic acid, adenosine 5′‐monophosphate (5′‐AMP), inosine 5′‐monophosphate (5′‐IMP), succinic acid, citric acid, formic acid, pyroglutamic acid, malic acid, lactic acid, and phosphates such as Na^+^, Mg^2+^, K^+^, Ca^2+^, and Cl^‐^, and other small molecule taste compounds.

Flavor precursors including amino acids, reducing sugars, 5′‐IMP, and polyunsaturated fatty acids are all in a dynamic state of degradation during storage (Koutsidis et al., 2008; Meinert et al., 2009; Mungure et al., 2016; Tikk et al., 2006). The umami (translated from Japanese as “savory deliciousness”) has come to be understood as the “fifth basic taste sensation,” elicited by the common flavor enhancer monosodium glutamate (MSG) along with other amino acids and ribonucleotides (Wang et al., 2019). Free amino acids are important for the formation of aroma volatiles as well as taste characteristics. Different amino acid profiles could result in the generation of different taste (Williamson et al., 2014). The flavor precursors could react with other degradation products for the formation of components responsible for present meat taste during heating.

Amino acids, organic acids, nucleotides, and other small organic compounds could be important taste substances and flavor precursors for beef flavor. The change of these compounds under different stewing conditions still needs to be further studied, which could be of great significance to optimize the processing technology of beef broth.

The aims of this study were to investigate the effect of stewing process on the content of taste compounds in stewing beef broth. The selected important variables of stewing beef broth included stewing time and the amount of sucrose, and salt. The amino acids, 5′‐nucleotides, and organic acid were determined by high‐performance liquid chromatography (HPLC). The equivalent umami concentration (EUC) and taste activity value (TAV) of stewing beef broth samples were calculated and compared, and the contribution of each component to the overall taste was analyzed.

## 2 MATERIALS AND METHODS

### 2.1 Materials

The beef (knuckle) that was purchased in Yonghui market (Beijing, China) was cut into beef cuts of 3 cm × 3 cm × 3 cm and stored at (4 ± 0.5)^o^C. Salt was purchased in China National Salt Industry Group Co., Ltd. Sucrose was purchased in Beijing Yulixing Commercial Trade Co., Ltd. Blend oil was purchased in Jiali Grain and Oil (Shenzhen) Co., Ltd. The spices package was made in laboratory (*m*_pepper_: *m*_shallot_: *m*_cumin_: *m*_onion powder_: *m*_coriander powder_ = 4:7:3:9:1); the materials were supplied by Shandong Fufeng Fermentation Co., Ltd.

### 2.2 Chemicals

Durashell AA analysis kit contained internal standard and 17 kinds of amino acid standard, which was purchased from Agela Technologies (Tianjin, China). Oxalic acid, tartaric acid, lactic acid, citric acid, succinic acid, hydrochloric acid (HCl), disodium hydrogen phosphate dodecahydrate (Na_2_HPO_4_•12H_2_O), potassium dihydrogen phosphate (KH_2_PO_4_), phosphoric acid (H_3_PO_4_) (all AR grade), and malic acid (BR grade) were obtained from Sinopharm Chemical Reagent Co. (Shanghai, China). Guanosine 5′‐monophosphate (5′‐GMP) disodium salt hydrate, 5′‐IMP, 5′‐AMP, and cytidine 5′‐monophosphate (5′‐CMP) were purchased from Sigma‐Aldrich (St. Louis, Mo., U.S.A.). Methanol, sulfosalicylic acid, and acetonitrile (ACN) (all HPLC‐grade) were purchased from Fisher Scientific (Shanghai, China). The ultrapure water was purchased from Hangzhou Wahaha Group Co., Ltd. (Hangzhou, China). Buffer salt I (0.05 mol/L KH_2_PO_4_) was prepared by dissolving 6.80 g KH_2_PO_4_ in 1 L water. Buffer salt II (0.01 mol/L KH_2_PO_4_) was prepared by dissolving 1.36 g KH_2_PO_4_ in 1 L water and then adjusted to pH 2.8 by 1 mol/L H_3_PO_4_.

### 2.3 Preparation of stewing beef broth sample

Beef cuts, salt, sucrose, spices package, blending oil, and water were added in an electric cooker (DGD32‐32BG, Tonze, Guangdong, China) and stewed in a nutrient soup model. Then, the beef broth samples with different stewing conditions were cooled to room temperature. The superficial fat was removed to get the beef broth samples. The stewing time and the addition of different amounts of all affected the overall taste of the stewed beef broth.

Preparation of beef broth samples with different stewing time: The 100 g beef cuts and 150 g water were added in the electric cooker (five copies) and stewed in a nutrient soup mode at 100°C. Six gradients (0, 2, 3, 4, 5, and 6 hr) were selected for stewing, separately.

Preparation of beef broth samples with the different salt addition: The 100 g beef cuts and 150 g water were added in the electric cooker (five copies), added 1.25, 1.50, 1.75, 2.00, and 2.50 g salt to the electric cooker, respectively, and stewed in a nutrient soup model with the stewing temperature of 100°C and stewed for 3 hr.

Preparation of beef broth samples with different sucrose addition: The 100 g beef cuts and 150 g water were added in an electric cooker (five copies), added 1.25, 1.50, 1.75, 2.00, and 2.50 g sucrose to the electric cooker, respectively, and stewed in a nutrient soup model with the stewing temperature of 100°C and stewed for 3 hr.

### 2.4 Preparation of beef broth samples with different stewed formulas

Six kinds of stewing methods with different materials are as follows: Stewed blend oil (s‐o) included 27 g blend oil and 150 g water; stewed spices (s‐s) included 1.50 g spices package and 150 g water; stewed beef blank group (s‐b‐b) included 100 g beef cuts and 150 g water; stewed beef broth blank group with spices (s‐b‐b‐s) included 1.50 g spices package, 100 g beef cuts, and 150 g water; stewed beef broth optimal group (s‐b‐o) (the optimum formula of stewed beef broth was based on the sensory evaluation experiment in the laboratory) included 1.50 g spices package, 1.80 g sucrose, 2.125 g salt, 100 g beef cuts, and 150 g water; stewed beef broth optimal group with blend oil (s‐b‐o‐o) included the materials of “s‐b‐o” with 27 g blend oil. These 6 samples were stewed in the nutrient soup model at 100°C for 3 hr.

### 2.5 Amino acid analysis

A Durashell AA Kit (Kong et al., 2017) was used to analyze amino acid in beef broth samples stewed under different conditions. The beef broth samples were cooled to room temperature, the meat was removed, and the supernatant was taken and placed in a refrigerator at 4°C for 12 hr. The broth supernatant was taken after removing oil and fat. The beef broth samples (6 g) were centrifuged for 10 min at 4°C and 6,010 g relative centrifugal force (RCF). The supernatant was taken, and 3.5 ml 20% (*m/V*) sulfosalicylic acid was added to the samples. The sample solution was volumed to 10 ml with 0.1 mol/L HCl. The TFAA concentration in samples was about 1–2 mg/ml. After filtered through 0.22‐µm nylon filter membrane (Cleman, Beijing, China), 500 μl beef broth sample and 50 μl internal standard solution were put into a 2 ml injection vial with pipette (Eppendorf, Hamburg, German) and then mixed by a vortex mixer (DragonLab, Beijing, China). The mixed sample solution was analyzed by Agilent 1,260 HPLC with a DAD detector (Agilent Corp., Karlsruhe, Germany). The 17 amino acids, including Asp, Glu, Ser, Pro, Gly, Thr, Ala, Val, Met, Ile, Phe, Lys, Leu, Arg, His, Tyr, and Cys‐Cys, were used as standard to determine the amino acids in beef broth. The concentration of Cys‐Cys was 0.014–0.341 mol/L, other 16 amino acids were between 0.027 and 0.682 mol/L, with an internal standard solution of Nva and Sar. Amino acids were detected by HPLC with the Durashell AA column (150 mm × 4.6 mm i.d., 3 µm) purchased from Agela Technologies (Tianjin, China). The mobile phase of the HPLC used to analyze amino acid was consisted of mobile phase A (12.5 mM Na_2_HPO_4_ and Na_2_B_4_O_7_ dissolved in ultrapure water, adjusted pH to 8.2) and mobile phase B (45% methanol, 45% acetonitrile, and 10% ultrapure water). The mobile phase gradient method was 6%–10% B from 0 to 6 min, hold 10% B from 6 to 8 min, 10%–16% B from 8 to 10 min, 16%–40% B from 10 to 23 min, 40%–50% B from 23 to 40 min, hold 100% B from 31 to 34 min, hold 6% B from 35 to 38 min. The column temperature was at 45°C, the mobile phase flow rate was 1.6 ml/min, and the UV detection was 338 nm and 262 nm for amino acid analysis. Each sample was analyzed three times.

### 2.6 The 5′‐nucleotides analysis

After refrigerating (4°C, 12 hr) and removing oil and fat, 5 g of the beef broth sample supernatant was centrifuged for 10 min at 4°C and 6,010 g relative centrifugal force (RCF). After being diluted 20 times, the samples were filtered through 0.22‐µm nylon filter membrane; then, 4 5′‐nucleotides in the samples were analyzed by Thermo U3000 UPLC (Thermo Fisher Scientific Inc., USA), and the data were collected, processed, and analyzed by Chromeleon software. The conditions of 5′‐nucleotide detection were as follows: column (Venusil XBP C_18_(4.6 mm × 250 mm, 5 μm) purchased from Agela Technologies (Tianjin, China); mobile phase was Buffer salt I (0.05 mol/L KH_2_PO_4_) and methanol (5:95, *V*/*V*), equal gradient elution, with a flow rate of 1ml/min; the column temperature was 25°C, the injection volume was 20 μl, and the UV detection wavelength was 254 nm. The mixed 5′‐nucleotide external standard (5′‐IMP, 5′‐GMP, 5′‐AMP, and 5′‐CMP) was prepared with ultrapure water and diluted into 7 gradients. According to the concentration range of four 5′‐nucleotides in the samples, the concentration of mixed external standard was 66.700, 40.000, 33.300, 20.000, 10.000, and 2.000 µg/ml, respectively. Each sample was analyzed three times.

### 2.7 Organic acid analysis

The method of sample preparation was the same as the "2.6. The 5′‐nucleotides analysis." The conditions of organic acid detection were as follows: The type of the column and the column temperature, the mobile phase elution gradient, the flow rate and the injection volume were all the same as the setting of "2.6. The 5′‐nucleotides analysis." The mobile phase was Buffer salt II 0.01 mol/L KH_2_PO_4_ (pH = 2.8) and methanol (5:95, *V*/*V*), and the UV detection wavelength was 205 nm. The mixed external standard included 4 mg/ml malic acid and citric acid, 2 mg/ml lactic acid, tartaric acid and succinic, and 1 mg/ml oxalic acid, and then diluted to 7 gradients. According to the concentration range of 6 organic acids in the samples, 7 standard mixed solutions were selected as follows: malic acid and citric acid: 2.667, 2.000, 0.800, 0.400, 0.133, and 0.067 mg/ml; lactic acid, tartaric acid, and succinic acid: 1.333, 1.000, 0.400, 0.200, 0.1000, 0.067, and 0.033 mg/ml; oxalic acid: 0.667, 0.500, 0.200, 0.100, 0.050, 0.033, and 0.017 mg/ml, respectively. Each sample was analyzed three times.

### 2.8 TAV analysis

TAV is the ratio of the taste‐active compounds in the samples to their taste threshold (Schlichtherle‐Cerny & Grosch, 1998). In this work, the taste threshold of flavor compounds (generally measured in water or in a simple matrix) was selected from Kato′s study (Kato et al., 1989). When the TAV was more than 1, it was considered that the compounds contributed to the taste. On the contrary, it did not contribute to the taste. The calculation formula of TAV is as follows:

$$\text{TAV}=C_{1/}C_{2}$$

*C*_1_ is the concentration of the flavor compound(mg/L), and *C*_2_ is the taste threshold (mg/L).

### 2.9 The equivalent umami concentration (EUC) analysis

The EUC value refers to the intensity of the umami taste produced by the synergistic action of the umami amino acids (Asp and Glu) and the 5′‐nucleotide (5′‐IMP, 5′‐GMP, and 5′‐AMP) in the dishes, which is equal to the concentration of the sodium glutamate (MSG) (Chen & Zhang, 2007). The umami taste produced by the 100 g samples could be equal to the mass of MSG, which is calculated as follows:

$$\text{EUC}\;\left(\;g/l00\;g\right)=\Sigma a_{i}\times b_{i}+\;1218\left(\Sigma a_{i}\times b_{i}\right)\left(\Sigma a_{j}\times b_{j}\right)$$

According to the formula, *a_i_* is the concentration of umami amino acid  (g/100 g) Asp and Glu; *b_i_* is the relative of umami coefficient (RUC) of each umami  amino acid relative to MSG, in which Asp (0.077) and Glu (1); *a_j_* is the concentration of umami 5′‐nucleotides (g/100 g), including 5′‐IMP, 5′‐GMP, and 5′‐AMP; *b_j_* is the RUC of umami 5′‐nucleotides relative to 5′‐IMP, in which 5′‐IMP (1), 5′‐GMP is (2.3), and 5′‐AMP (0.18).

### 2.10 Statistical analysis

The data processing of this experiment was carried out by SPSS (version 17.0, SPSS Inc., Chicago, IL, USA). Significance analysis between samples was performed by one‐way ANOVA and Duncan′s multilevel test (*p* <0.05) (Zheng et al., 2016). All statistical analyses were performed in triplicate, and the results were expressed as mean ± *SD*.

## 3 RESULTS AND DISCUSSION

### 3.1 Analysis of the contents of general components

For the selected beef cut (knuckle) samples, the general components of the samples were determined. Knuckle is located on the inside of the hind legs of the cattle, and it is round and smooth in appearance, thin and tender in meat, and thick in fiber, which is suitable for both stewing and stir‐frying. China national standard methods (GB/T 22221‐2008, GB 5009.3‐2016, and GB 5009.5‐2010) were used to analyze the contents of protein, moisture, fat, and sugars (fructose, glucose, sucrose, maltose, and lactose) in samples. The content of moisture in knuckle was 70.80 g/100 g, which was the main component of beef, followed by protein content (21.9 g/100 g). Knuckle contained only a small amount of ash (1.00 g/100 g) and fat (1.10 g/100 g), and the content of the five sugars detected in the sample was less than 0.10%. From previous studies, beef has high contents of biological value protein, VB_6_, VB_12_, low fat, and rich in amino acids and long‐chain omega‐3 polyunsaturated fats, compared with pork; the amino acids in beef protein are more essential to human essential amino acids (Bendtsen et al., 2019; Pereira & Vicente, 2013).

### 3.2 The content of tastes in stewed beef broth with different stewing time.

**Figure 1:**
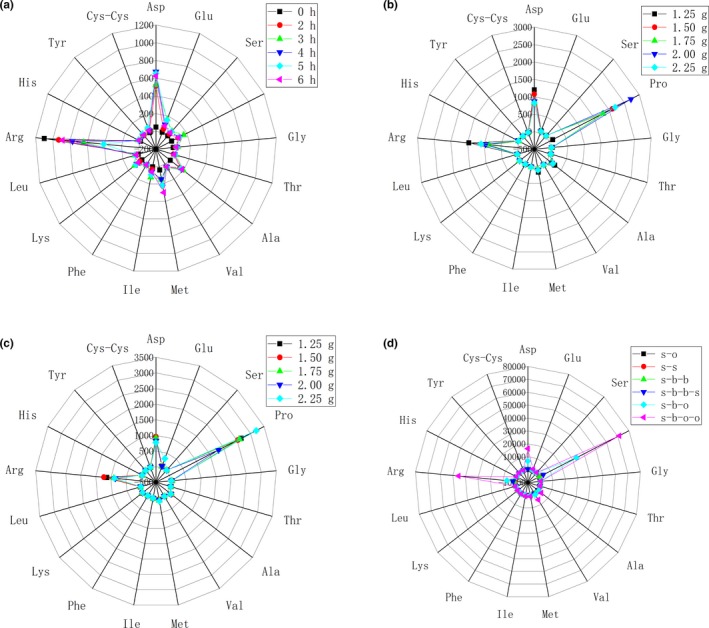
The contents of 17 amino acids in stewed beef broth with different conditions (a, stewing time; b, the amount of salt addition; c, amount of sucrose addition; d, different formula (stewed blend oil (s‐o); stewed spices (s‐s); stewed beef broth blank group (s‐b‐b); stewed beef broth blank group with spices (s‐b‐b‐s); stewed beef broth optimal group (s‐b‐o); stewed beef broth orthogonal optimal experimental group with blend oil (s‐b‐o‐o))

**Figure 2:**
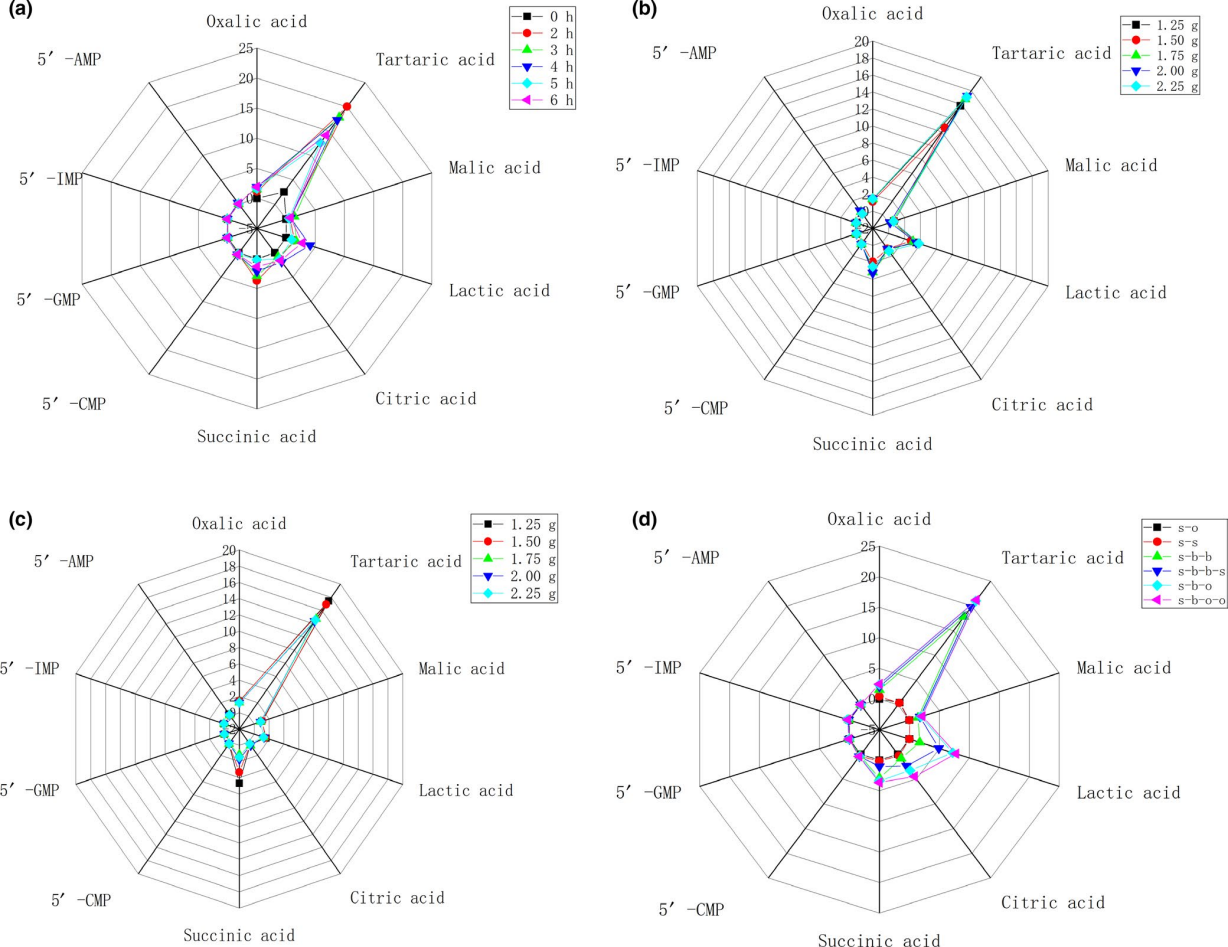
The contents of six organic acids (malic acid, citric acid, lactic acid, tartaric acid, oxalic acid, and succinic acid) and four 5′‐nucleotides (5′‐IMP, 5′‐GMP, 5′‐AMP, and 5'‐CMP) in stewed beef broth with different conditions (a, stewing time; b, the amount of salt addition; c, amount of sucrose addition; d, different formula)

Figure 1 shows the contents of 17 amino acids in stewed beef broth with different conditions. The contents of 6 organic acids and four 5′‐nucleotides are shown in Figure 2. Seventeen free amino acids could be classified into four categories: umami, sweet, bitter, and no‐taste amino acids (Liu et al., 2015; Zhou et al., 2016). In Figure 1, 14 kinds of free amino acids were detected in raw beef. The total contents of umami and sweet free amino acids were 52.83 and 17.77 μg/g in raw beef, respectively, which were much lower than those in beef broth after stewing. However, the contents of total bitter taste free amino acids in raw beef were higher, such as the content of Arg in raw beef, which was the highest in all 6 samples with different stewing time. Qi et al. (2018) determined the taste‐active compounds in stewed Chinese yellow‐feather chicken soup. The content of total free amino acids was 258.52 μg/g in the raw chicken meat, and stewing significantly reduced the concentration of free amino acids in chicken meat. In this work, with the increase in stewing time, the contents of umami amino acids (Asp and Glu) increased during stewing for 5 hr (804.42 μg/g) and then decreased. The content of Asp increased to the maximum value at stewing for 4 hr (673.24 μg/g). The content of Glu increased to the maximum value at stewing for 5 hr (151.70 μg/g). Cao et al. (2013) studied the sensory evaluation of chicken soup with different cooking time. Their results showed that the flavor of chicken soup increased obviously during cooking from 2 to 4 hr, but decreased obviously with the increase in boiling time more than 4 hr. Pro and Ala were the main sweet amino acids in beef broth, which reached the maximum at 3‐hr stewed beef broth, which were 158.62 and 197.13 μg/g, respectively, and then decreased. The total amount of sweet amino acid reached the maximum at stewing for 3 hr (519.75 μg/g). Kaur et al. (2014) reported that cooking conditions had a greater impact on the degradation of beef protein. Long‐term stewing could lead to incomplete degradation of small molecular weight (<10 kDa) peptides. In this work, the total contents of bitter amino acids decreased from 1,220.55 to 951.11 μg/g at stewing for 0 to 5 hr, and then increased to the maximum value of 1,422.71 μg/g at stewing for 6 hr.

Organic acids and their monosodium succinate and disodium salts have an umami taste or enhancing flavor. 5′‐AMP, 5′‐IMP, and 5′‐GMP have been identified as flavor substances and widely used in food industry (Shoji et al., 2016). The content of succinic acid continued to significantly (*p* <0.05) decrease with the increase in stewing time at 2 to 6 hr, from 3.67 μg/g to 0.18 μg/g. It indicated that high‐temperature and long‐term heating lead to the degradation of succinic acid.

Qi et al. (2018) studied the taste‐active compounds in stewed Chinese yellow‐feather chicken. The results showed that the contents of taste components such as free amino acids, 5′‐nucleotides, and minerals significantly decreased with the increase in stewing time. 5′‐IMP was the major umami compounds in stewed yellow‐feather chicken meat and decreased significantly during stewing. Figure 2a shows the contents of four 5′‐nucleotides in stewing beef broth with different stewing time. In Figure 2a, the contents of four 5′‐nucleotides in raw beef were significantly lower than that in stewed beef broth (*p* <0.05), which showed that stewing could increase the contents of 5′‐nucleotides in beef broth (Yue et al., 2016). The maximum content of total 5′‐nucleotides was 0.76 μg/g at stewing beef for 4 hr. The maximum content of 5′‐GMP and 5′‐AMP was 0.08 μg/g and 0.27μg/g at stewing beef for 4 hr, respectively.

### 3.3 The content of tastes in stewed beef broth with different salt addition

Figure 1b and Figure 2b show the contents of taste compounds in stewed beef broth with different salt addition. In Figure 1b, the maximum content of total amino acid in stewed beef broth was 5,088.76 μg/g at 2 g salt addition. Compared with Figure 1a, the content of total amino acid in stewed beef broth without salt addition was only 2,448.62 μg/g. The addition of salt could promote the release of amino acid in beef stewing. The maximum content of total sweet amino acid in stewed beef broth was 2,930.11 μg/g at 2 g salt addition. The contents of Asp, Pro, and Arg were higher in 17 amino acids. The maximum content of Asp and Arg was 1,200.55 and 1,461.10 μg/g at 1.25 g salt addition, respectively. The maximum content of Pro was 2,717.60 μg/g at 2.00 g salt addition. With the increase in salt addition, the content of umami amino acid significantly decreased in stewed beef broth. The results showed that low concentration of NaCl promoted the release of nonvolatile flavor components. The Na^+^ is the salty targeting base in salt (NaCl). The Cl^‐^ is the flavoring base, and proper salt is added in monosodium glutamate (MSG) solution to make it taste more umami (Li et al., 2010).

In Figure 1b and Figure 2b, the maximum content of succinic acid in stewed beef broth was 3.24 μg/g at 2 g salt addition. The NaCl could increase the release of umami organic acid, produced more umami enhancer sodium succinate disodium salt, and improved the delicacy of dishes (Kawasaki et al., 2016). The content of 5′‐GMP decreased with the increase in salt addition from 1.75 to 2.25 g. The maximum content of total nucleotides was 1.02 μg/g at 2.00 g salt addition. Compared with the beef stewed broth for 3 hr without salt in Figure 2a, the maximum content of total nucleotides was 0.76 μg/g without salt addition. The results showed that the contents of flavor nucleotides increased significantly after salt addition, which indicated that the addition of salt was beneficial to the release of flavor nucleotides from stewed beef broth (Kong et al., 2017).

### 3.4 The content of tastes in stewed beef with different sucrose addition

Figure 1c and Figure 2c show the contents of taste compounds in stewed beef broth with different sucrose addition. The maximum content of umami, sweet, and total amino acid in stewed beef broth was 1085.10, 3367.48, and 5595.20 μg/g at 2.25 g sucrose addition, respectively. The content of total bitter amino acid was higher at 1.25 and 1.50 g sucrose addition and decreased with the increase in sucrose addition. Compared with the stewed beef broth with salt addition in Figure 1b, the maximum content of total amino acid was 5088.76 μg/g. The addition of sucrose had a greater effect on the content of total amino acids in stewed beef broth than the addition of salt. It indicated that the stewed beef broth with sucrose also had a significant effect on the overall taste of stewed beef broth.

The content of organic acid in stewed beef broth with different sucrose addition is shown in Figure 2c. The content of succinic acid showed a decreasing trend with the increase in sugar content, and the maximum content was 4.69 μg/g when the sugar addition was 1.25 g. Compared with Figure 2a and Figure 2b, the stewed beef broth was stewed for 3 hr and the salt was added; the maximum content of succinic acid was 2.92 and 3.24 μg/g, respectively, which indicated that added sucrose in stewed beef broth could promote the release and dissolution of umami organic acids (Schlichtherle‐Cerny & Grosch, 1998).

Sweetness and umami have a synergistic effect, and the stewed dishes often describe their delicacy with umami and sweet taste (Shoji and others 2016). The contents of 5′‐IMP and 5′‐AMP were obtained at the maximum values when the amount of sucrose was 1.25 g, which were 0.10 and 0.26 μg/g, respectively. With the increase in sucrose addition to more than 1.75 g, the content of nucleotide was not significantly different (*p* > 0.05). Compared with Figure 2a, the contents of nucleotides in stewed beef broth increased significantly after adding sucrose, indicating that the addition of sucrose was beneficial to the release of flavor nucleotides in stewed beef (Gurikar, Lakshmanan, Gadekar, Sharma, & Anjaneyulu, 2014).

### 3.5 The content of tastes in stewed beef broth with different formulas

Figure 1d and Figure 2d show the contents of taste compounds in stewed beef broth with different formula. Compared with the s‐b‐b, the addition of spices package to the stewed beef broth (s‐b‐b‐s) promoted the release of umami and sweet amino acid in the beef broth. The contents of Asp, total sweet amino acid, and total amino acid in s‐b were 535.22, 519.74, and 2464.47 μg/g, respectively, and the contents of those compounds in stewed beef after adding spices (s‐b‐b‐s) were 546.00, 4004.99, and 6694.65 μg/g. It indicated that spices addition was beneficial to promote the release of amino acid in beef broth. The highest contents of umami, sweet amino acid, and total amino acid were 907.67, 2930.11, and 5088.76 μg/g in stewed beef broth with salt addition, and 1085.10, 3367.48, and 5595.20 μg/g with sucrose addition. The contents of those in stewed beef broth optimal group (s‐b‐o) were 7008.53, 34007.67, and 49282.82 μg/g, respectively, which was far higher than that content with salt addition and sucrose addition. The contents of umami, sweet amino acid, and total amino acid in the stewed beef broth optimal group with oil (s‐b‐o‐o) were also significantly increased to 17452.31, 75692.62, and 147228.60 μg/g, which were significantly different from s‐b‐o (*p* < .05). It indicated that the addition of blend oil could promote the release of amino acid in stewed beef broth.

Figure 2d shows the contents of organic acids in stewed beef with different formulas. None succinic acid was detected in the sample of s‐o. The content of succinic acid in s‐s was 0.152 μg/g. The stewed spices package could also have umami taste (Zou et al., 2018). The content of succinic acid in s‐b‐o was 3.39 μg/g, which was significantly different from that in s‐b (2.92 μg/g) and s‐b‐s (1.03 μg/g). The content of total organic acid was significantly different between the s‐b‐o and the s‐b‐o‐o (*p* <0.05). It indicated that the addition of blend oil could promote the release of organic acids in stewed beef broth.

Figure 2d shows the contents of 5′‐nucleotides in stewed beef broth with different formulas. In Figure 2d, none of the nucleotides was detected in s‐o. The 5′‐IMP, 5′‐GMP, 5′‐AMP, and 5′‐CMP were detected in s‐s with the total content of 0.48 μg/g. The addition of spices could significantly increase the 5′‐nucleotides of stewed beef broth. Kranz et al. (2018) also added spices such as leeks, onions, celery, and cloves to make traditional beef soup Pot‐au‐Feu Broth and improve the taste of beef broth. Compared with the contents of nucleotides in s‐b‐b and stewed beef broth with only salt or sucrose addition, the content of 5′‐IMP and 5′‐GMP in the sample of s‐b‐o was significantly increased. The optimized beef broth sample contained more taste compounds.

According to the contents of taste compounds in stewed beef broth with different formulas, the contents of amino acid, nucleotides, and organic acids in stewed beef with blend oil (s‐b‐o) increased significantly. The content of total amino acid and total organic acid was significantly higher in s‐b‐o‐o than in s‐b‐o. The proper amount of blend oil was beneficial to the release of flavor substances in stewed beef broth.

### 3.6 The TAVs in stewed beef broth

The TAVs in stewed beef broth with different formulas are listed in Table 1. Although the content of some flavor substances was low, the lower threshold could make them to have an important contribution to the taste (Kong et al., 2017). The TAVs of Asp and Glu, Pro, and tartaric acid in s‐b‐b and s‐b‐b‐s were more than 1. With spices addition, besides Glu, the TAVs of taste compounds increased in stewed beef. It indicated that the contribution of taste substances in stewed beef increased with spices addition. The TAVs of Asp, Glu, Pro, Ala, Arg, and tartaric acid in s‐b‐o and s‐b‐o‐o were more than 1, which indicated that these compounds played a key role in the taste of stewed beef. Compared with the TAVs of s‐b‐o and s‐b‐o‐o, the TAV of 7 compounds (TAV > 1) increased with oil addition in stewed beef broth, including Asp, Glu, Pro, Ala, Val, Met, and Arg. The results showed that stewing with blend oil had a great influence on the release of flavor substances in stewed beef broth. Kong et al. (2017) measured and calculated the TAV of chicken soup and chicken enzymatic hydrolysate. The result showed that His had the highest TAV (2.60) among the 17 amino acids of chicken soup and 1.72 in chicken enzyme hydrolysate, but the TAVs of other amino acids were less than 1. The TAVs of lactic acid and succinic acid in chicken soup and chicken enzyme hydrolysate were 4.07, 5.36, 3.71, and 16.58, respectively, which was demonstrated that the two organic acids were the main flavor organic acids in the chicken soup and chicken enzyme hydrolysate. The TAVs of 5′‐nucleotide were less than 1, which may not contribute to the taste of chicken soup and chicken hydrolysate. Qi et al. (2018) detected and calculated the TAV of stewed Chinese yellow‐feather chicken soup. It was known that the TAVs of other flavor compounds except 5′‐IMP were less than 1, but they worked synergistically with 5′‐IMP to strongly enhance the umami taste.

**Table 1:** The TAVs of taste compounds in stewing beef broth about different formulas

Compounds	Taste threshold (mg/L)	TAV					
		s‐o	s‐s	s‐b‐b	s‐b‐b‐s	s‐b‐o	s‐b‐o‐o
Asp	300	ND	ND	1.74	1.82	22.14	54.274
Glu	50	ND	ND	3.27	1.97	7.36	23.401
Ser	1,500	ND	ND	0.05	0.02	0.09	0.29
Pro	3,000	ND	ND	0.05	1.22	11.05	23.767
Gly	1,300	ND	ND	0.04	0.04	0.03	0.272
Thr	2,600	ND	ND	0.02	0.02	0.02	0.076
Ala	600	ND	ND	0.33	0.43	1.04	5.68
Val	400	ND	ND	0.11	0.06	2.45	14.324
Met	300	ND	ND	0.5	0.64	0.6	3.097
Ile	900	ND	ND	0.14	0.01	0.03	0.66
Phe	900	ND	ND	0.01	0.01	0.12	0.2
Lys	500	ND	ND	0.22	0.03	0.08	0.485
Leu	1900	ND	ND	0.03	0.01	0.13	0.22
Arg	500	ND	ND	1.28	3.57	13.61	91.01
His	200	ND	ND	0.14	0.05	0.27	0.84
Tyr	ND	ND	ND	ND	ND	ND	ND
Oxalic acid	504	ND	0.001	0.003	0.005	0.005	0.004
Tartaric acid	15	0.03	0.03	1.19	1.40	1.41	1.32
Malic acid	496	ND	ND	0.003	0.004	0.004	0.004
Lactic acid	1,260	ND	ND	0.001	0.006	0.006	0.004
Citric acid	450	ND	0.001	0.002	0.007	0.01	0.005
Succinic acid	106	ND	0.001	0.03	0.02	0.03	0.01
5′‐GMP	125	ND	ND	ND	ND	ND	ND
5′‐IMP	255	ND	ND	ND	ND	ND	ND
5′‐AMP	500	ND	ND	ND	ND	ND	ND

Note

ND represents not detectable.

### 3.7 The EUC in stewed beef broth with different formulas

**Figure 3:**
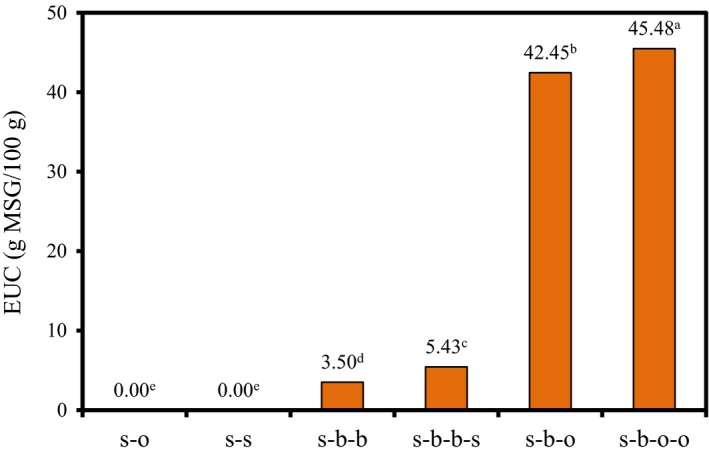
The EUC value of the stewed beef broth with different formulas. (Different superscripts between columns represent significant differences between cultivars (*p* < .05).)

The EUC value refers to the intensity of the umami taste produced by the synergistic action of the umami taste amino acids and the 5′‐nucleotide in the stewed beef, which is equivalent to the concentration intensity of the MSG (Chen & Zhang, 2007). Figure 3 shows the EUC value of stewed beef broth with different formulas. The EUC values of s‐o and s‐s were 0. The EUC values of s‐b‐b and s‐b‐b‐s were 3.50 and 5.43 g MSG/100 g, respectively. The results showed that spices addition could increase the content of flavor substances in stewed beef broth. Qi et al. (2018) determined and calculated that the EUC value of Chinese yellow‐feather chicken was 8.50 g MSG/100 g, which was higher than the EUC value (3.50 g MSG/100 g) of stewed beef broth (s‐b‐b) in this work. The EUC values of the s‐b‐o and s‐b‐o‐o were 42.45 and 45.48 g MSG/100 g, respectively, which was significantly higher than the s‐b‐b and s‐b‐b‐s. This indicated that the contents of flavor substances in stewed beef broth were higher after adding the blend oil and made the beef broth tasted more umami.

## 4 CONCLUSIONS

The 17 amino acids, 6 organic acids, and 4 5′‐nucleotides in beef broth under different stewing conditions were determined by HPLC. The TAVs and EUC values were also calculated and compared. The addition of salt, sucrose, blend oil, and spices package to the stewed beef increased the release of taste‐active compounds in the beef broth. The contents of four 5′‐nucleotides in raw beef were significantly lower than that in stewed beef broth, which showed that stewed could increase the contents of 5′‐nucleotides in beef broth. The contents of flavor nucleotides increased significantly after salt addition. The addition of spices, salt, and sucrose was beneficial to promote the release of amino acid in beef broth. The highest contents of umami, sweet amino acid, and total amino acid were 907.67, 2930.11, and 5088.76 μg/g in stewed beef broth with salt addition, and 1085.10, 3367.48, and 5595.20 μg/g with sucrose addition. The contents of those in stewed beef broth optimal group (s‐b‐o) were 7008.53, 34007.67, and 49282.82 μg/g, respectively, which was far higher than that content with salt addition and sucrose addition. The content of total amino acid and total organic acid was significantly higher in s‐b‐o‐o than in s‐b‐o. The proper amount of blend oil was beneficial to the release of flavor substances in stewed beef broth.

The EUC value of the stewed beef blank group (s‐b‐b) was 3.50 g MSG/100 g. With the spices addition, the EUC value of s‐b‐o was 42.45 g MSG/100 g. The addition of spices could significantly increase the EUC of stewed beef broth. The TAVs of 8 compounds were more than 1 in the sample of s‐b‐o‐o, including Asp, Glu, Pro, Ala, Val, Met, Arg, and tartaric acid. These 8 compounds contribute more to the taste of stewed beef broth.

## CONFLICT OF INTEREST

The authors declare that they have no conflict of interest.

## ETHICAL APPROVAL

This study does not involve any human or animal testing.
